# Graphene Oxide Enhances Chitosan-Based 3D Scaffold Properties for Bone Tissue Engineering

**DOI:** 10.3390/ijms20205077

**Published:** 2019-10-13

**Authors:** Sorina Dinescu, Mariana Ionita, Simona-Rebeca Ignat, Marieta Costache, Anca Hermenean

**Affiliations:** 1 Department of Biochemistry and Molecular Biology, University of Bucharest, Splaiul Independentei 91-95, 050095 Bucharest, Romania; simona.ignat@unibuc.ro (S.-R.I.); marieta.costache@bio.unibuc.ro (M.C.); 2 Advanced Polymer Materials Group, University Politehnica of Bucharest, Gh. Polizu 1-7, 011061 Bucharest, Romania; mariana.ionita@polimi.it; 3 Institute of Life Sciences, Vasile Goldis Western University of Arad, 86 Rebreanu, 310414 Arad, Romania; anca.hermenean@gmail.com

**Keywords:** graphene oxide, biocompatibility, bone tissue engineering, human adipose-derived stem cells, osteogenesis

## Abstract

The main goal of bone tissue engineering (BTE) is to refine and repair major bone defects based on bioactive biomaterials with distinct properties that can induce and support bone tissue formation. Graphene and its derivatives, such as graphene oxide (GO), display optimal properties for BTE, being able to support cell growth and proliferation, cell attachment, and cytoskeleton development as well as the activation of osteogenesis and bone development pathways. Conversely, the presence of GO within a polymer matrix produces favorable changes to scaffold morphologies that facilitate cell attachment and migration i.e., more ordered morphologies, greater surface area, and higher total porosity. Therefore, there is a need to explore the potential of GO for tissue engineering applications and regenerative medicine. Here, we aim to promote one novel scaffold based on a natural compound of chitosan, improved with 3 wt.% GO, for BTE approaches, considering its good biocompatibility, remarkable 3D characteristics, and ability to support stem cell differentiation processes towards the bone lineage.

## 1. Introduction

In the area of regenerative medicine, there is a constant need for the development of novel biomaterials with adaptive properties which are able to efficiently support the repair or regeneration of a damaged tissue. After implantation, these materials will be part of the local tissue repair process, together with the cells and the signaling molecules that mediate tissue self-renewal. In particular, the main goal of bone tissue engineering (BTE) is to refine and repair major bone defects based on bioactive biomaterials with distinct properties that can induce and support the formation of bone tissue. Graphene and its derivatives are nanomaterials with proven pro-osteogenic effects [1,2,3]. Recently, bi- and tridimensional scaffolds with a graphene basis have been proposed as templates for applications in the field of BTE. Graphene (a monolayer of carbon), and its derivatives such as graphene oxide (GO), offer a set of outstanding physical-chemical properties, making them the optimal choice for BTE [4].

Unlike other forms of graphene materials, GO is vastly oxidized, exhibiting hydroxyl and epoxide functional groups on its surface while carboxyl functional groups are present on its plane edges [5]. The presence of functional groups on the basal surface and plane edge of GO generates a hydrophilic behavior and improved solubility in aqueous suspensions. Furthermore -COOH and -OH groups facilitate the formation of bond and non-bond interactions with various materials, e.g., biopolymers, and thus hinder aggregation of GO sheets. Conversely, the formation of bond or non-bond interactions is critical in defining the functional, morphological, and mechanical features of a composite material [5].

Graphene and its derivatives have an overall positive impact on material biocompatibility and on cell adhesion to the substrate. Numerous studies have reported a low cytotoxic effect of GO that can be reflected by a decrease in cell viability [6,7]. In fact, cell response varies depending on the type of graphene derivative and the concentration included in the material’s composition. In this context, it was shown that including GO in a material generates a good interaction between the cellular component and the surface of the material. The GO surface presents a series of chemical groups such as carboxylate, hydroxyl, carboxyl, and carbonyl groups, which increase the interaction of the cellular proteins via some stable hydrogen bonds. Therefore, graphene and its derivatives favor optimal cytoskeleton development and promote cell adhesion in scaffolds [8]. Good cell adhesion is one of the first conditions for a successful differentiation process. GO ensures cell survival, growth, proliferation, and activation of molecular pathways, stimulating the mammalian cells to generate a bone-specific extracellular matrix [9].

Moreover, biomaterials that include GO have been used over the years as substrates for osteogenic differentiation. The chemical, physical, and mechanical proprieties of GO improve the structure of the material and certify the adhesion, proliferation, and differentiation of mammalian cells [10]. When GO-based scaffolds were used in osteogenic differentiation processes, higher expression of osteogenic markers (such as Runt-related transcription factor 2 (Runx2), osteocalcin (Ocn), and alkaline phosphatase) was reported [11]. Scaffolds with GO derivatives have also proven to be effective in promoting the osteogenic differentiation in vivo. They can promote osteogenesis and osteointegration when implanted at the injured site and further carry out the reparation of the bone defect [12]. Although the molecular interaction between GO and the cells is not yet completely understood, the high potential of GO is continuously being explored, especially for applications in the field of BTE.

Human adipose-derived stem cells (hASCs) have been recently used in numerous regenerative medicine approaches due to their easy harvesting, mesenchymal origin, and potential for differentiation. Several studies have shown the ability of hASCs to differentiate towards the bone lineage in vitro or to contribute to bone formation and repair in vivo. Nevertheless, the association between GO properties and the advantages of hASCs for bone development has been insufficiently explored [13,14].

Therefore, the aim of this study was to thoroughly evaluate the properties, biocompatibility, and potential of a newly developed chitosan scaffold (CHT) improved with 0.5–3% GO to support the bone differentiation process both in vitro and in vivo. We hereby used a complex approach to investigate the hASC response to the material, as well as the osteogenic differentiation to bone-like tissue both at gene and protein levels.

## 2. Results

### 2.1. Assessment of CHT/GO Composite Cytocompatibility

CHT/GO materials were evaluated for cytocompatibility against hASCs during one week of in vitro culture in standard conditions. After cells were embedded in the scaffolds, 3D cultures denominated as “bioconstructs” resulted. For more coherent data presentation, hASC/CHT/GO bioconstructs were denoted as “BCs” with different percentages (0.5–3 wt.%) of GO in the composite (BC0.5–BC3, respectively), and were compared to an hASC/CHT reference bioconstruct (BC) during the in vitro and in vivo biological studies.

**Figure 1:**
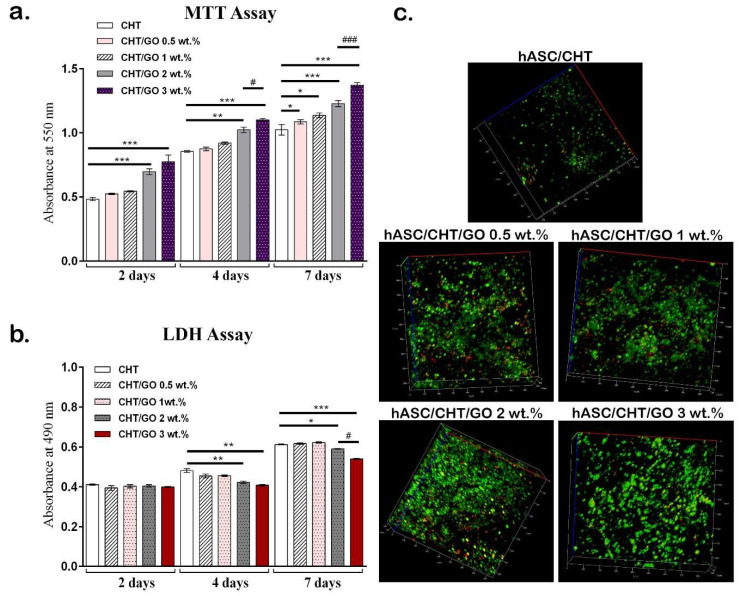
Cytocompatibility assessment of BC0.5–BC3 with human adipose-derived stem cells (hASCs). (**a**) Cell viability in contact with chitosan (CHT)/graphene oxide (GO) composites by 3-(4,5-dimethylthiazol-2-yl)-2,5-diphenyltetrazolium bromide (MTT) assay; (**b**) CHT/GO material levels of cytotoxicity on contact with hASC culture by lactate dehydrogenase (LDH) assay; (**c**) tridimensional reconstructions for BC0.5-BC3 and control showing live cells (green) and dead cells (red) after 7 days of culture resulted from Live/Dead assay and confocal microscopy analysis. */# *p* < 0.05; ** *p* < 0.01; ***/### *p* < 0.001.

Cytocompatibility assays results showed an overall good biocompatibility of all studied composites in relation to hASCs. Cell viability and proliferation were quantitatively evaluated by 3-(4,5-dimethylthiazol-2-yl)-2,5-diphenyltetrazolium bromide (MTT) (Figure 1a) and registered an increasing profile during one week of culture. Two days after the initiation of the 3D cultures, cells cultured in contact with high GO content (2 and 3 wt.%) materials displayed a statistically significant higher viability (*p* < 0.001) than the ones cultured in contact with CHT control or low GO content (0.5 and 1 wt.%). This statistical significance was also observed after 4 days of culture, where cells exposed to higher GO content in the material also displayed an increased proliferation potential as compared to the control (*p* < 0.001). An important observation is that after 4 days of culture, a statistically significant difference appeared between BC2 and BC3 (*p* < 0.05), which can suggest an early positive effect of GO on hASC proliferation, proportional to the GO concentration used in the composite. These observations were also confirmed after 7 days of culture in standard conditions, when all the studied composites presented statistically significant differences in relation to the control.

Next, CHT/GO material cytotoxicity was measured by lactate dehydrogenase (LDH) assay during one week of culture (Figure 1b). All biomaterials showed a low level of cytotoxicity after 2 days of culture in standard conditions. Four days after seeding, the levels of released LDH remained constant for BC2 and BC3, whereas a slight increase in LDH level was registered for BC0.5 and BC1, as well as for the BC control. This difference between BC0.5–BC1 and BC2–BC3 was statistically significant (*p* < 0.01). This trend was also observed after 7 days of culture, when BC0.5–BC1 registered similar cytotoxicity levels as the BC reference, whereas increasing the GO concentration to 3 wt.% led to a statistically significant decrease in the percentage of dead cells (*p* < 0.001) as compared to the control.

LiveDead assay confirmed the quantitative MTT and LDH results, showing a strong positive ratio between live (green) and dead (red) cells. Figure 1c shows 3D reconstructions obtained by confocal microscopy of all four bioconstructs versus the BC reference. Interestingly, the amount of cells increased proportionally to GO concentration in the scaffolds, suggesting a positive GO influence on cell proliferation.

Although many studies indicate that the addition of GO in the composition of the materials generally leads to an increase in cytotoxicity [6,7], others report that GO can have a positive or no effect on cell viability [15,16]. Overall, scaffolds containing GO display good biocompatibility and may favor cell proliferation. Our results obtained on the BC0.5–BC3 constructs support this observation.

### 2.2. Evaluation of hASC Morphology and Cell Cytoskeleton Organization in BC0.5–BC3

**Figure 2:**
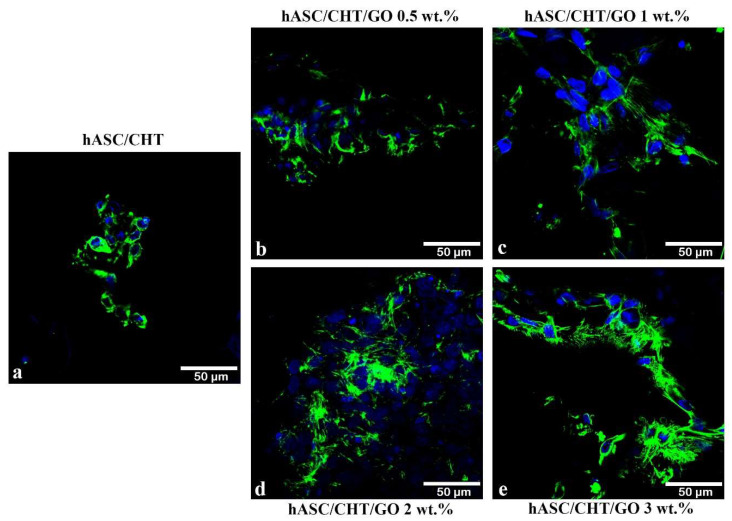
Actin filaments developed by hASCs after 48 h of contact with CHT/GO (**b–e**) and the CHT reference (**a**), as shown by confocal microscopy. Scale bar 50 μm. Actin filaments are shown in green (phalloidin-fluorescein isothiocyanate (FITC)) and cell nuclei are shown in blue (DAPI).

In the case of three-dimensional BC0.5-BC3, F-actin filaments were highlighted by phalloidin- fluorescein isothiocyanate (FITC) staining and confocal microscopy visualization 48 h after the cells were put in contact with the scaffolds. A strong tendency for better cell adhesion dependent on the GO content in the structure of each material was observed (Figure 2). In the case of BC control, hASCs did not develop a fusiform morphology and retained a rounded shape, without the presence of long actin filaments (Figure 2). When adding 0.5 wt.% GO to the scaffold’s composition, the actin filaments started to develop (Figure 2b) and the fusiform phenotype was achieved starting with 1 wt.% GO. The best-developed cytoskeleton was highlighted in the case of hASCs grown in contact with CHT/ GO 3 wt.% (Figure 2e), which confirmed this scaffold was suitable for hASC adhesion.

Substrate adhesion is a crucial step for many cellular functions such as proliferation, protein synthesis, or the formation of mineral deposits. Kalbacova et al. [16] reported that bone marrow mesenchymal stem cells (BM-MSCs) cultivated on SiO_2_ substrates formed homogeneously distributed focal adhesions at the periphery of cells [13]. Also, investigation of the interaction between hASC and GO-coated films determined that cells adhere better in the presence of GO, as indicated by the large number of focal adhesions and correlation of the orientation of actin filaments with that of bands of vinculin, as compared to hASCs grown on glass reference substrates [17]. A number of other studies [18] also indicated that GO promotes the adhesion of mesenchymal stem cells to the substrates on which they were grown.

Based on the cytocompatibility results and observations from the evaluation of hASCs morphology in contact with CHT/GO 0.5–3 wt.%, it was concluded that cells displayed a distinct behavior when exposed to high GO-content scaffolds (2–3 wt.%), as compared to low GO-content materials (0.5–1 wt.%). Therefore, representative composites were chosen for further in depth studies, namely CHT/GO 0.5 wt.% (BC0.5) and CHT/GO 3 wt.% (BC3), to be compared to the pure CHT control (BC).

### 2.3. CHT/GO Materials Characterization by MicroCT

**Figure 3:**
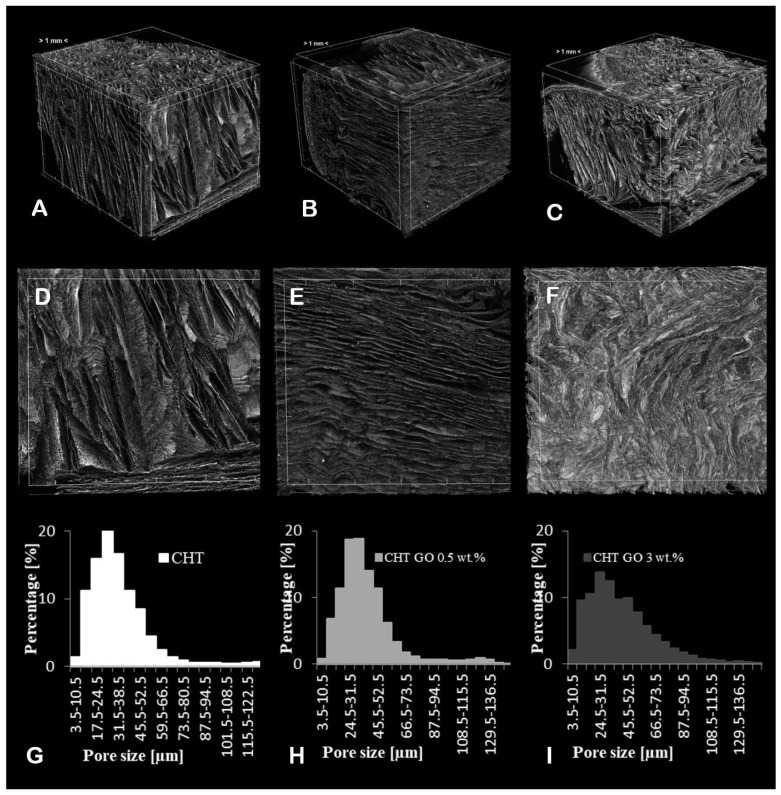
CHT/GO material characterization by MicroCT. Three-dimensional renderings of CHT (**A**), CHT/0.5 wt.% GO (**B**), and CHT/3 wt.% GO (**C**), with complementary cross-sections (**D**,**E**,**F**) and pore size distributions (**G**,**H**,**I**). The overall scale bar is 1 mm.

The CTVox 3D rendering of CHT, CHT/0.5 wt.% GO, and CHT/3 wt.% GO volumes in the scaled cutting box and complementary 2D slice depicting the composite dominant morphology were assessed (Figure 3). The scale was kept constant for all samples; the distance in between two checkmarks was fixed at 1 mm. All samples exhibited highly porous architecture, with a high degree of anisotropy regarding pore orientation and increased interconnected porosity. With respect to the total sample volume, closed porosity amounted to less than 0.1%. 

In Table 1, total porosity, structure thickness, and specific surface were summarized. Specific surface is expressed as fraction between object surface and object volume; thus, it was strongly altered by the porosity extent. Upon addition of 0.5 wt.% GO nanofiller to the CHT matrix, total porosity and specific surface values decreased, in good agreement with previously reported data on polymer-GO composites [19]. By further increasing to a 3 wt.% GO amount, the total porosity of CHT/GO composite scaffold was heightened to 76%, which unquestionably influences its specific surface in a direct variation.

Further we looked at the structure thickness (St.Th), a measure that encompasses the thickness of all the objects in a sample (weighted average of material wall). There was an important increase of St.Th. occurring after GO addition in the polymer matrix, indicating the formation of pores with thicker and well defined walls able to better support cell attachment.

From a different perspective, one could argue that GO increases T.Po and St.Th while keeping the St.Th unaltered through a pore shaping mechanism. In Figure 3A,D, CHT pores featured random orientation and sharp edges. On the other hand, upon GO addition pore domains became more ordered (Figure 3B,E), and at the highest GO load pore walls were remodeled into sinuous frameworks (Figure 3C,F). Curved apertures displayed by the polymer composites provide greater surface areas, highest porosity in minimal volumes and the finest interfaces for cell migration and attachment.

Regarding pore size distributions in the three samples, an apparently similar pattern following a Gaussian right-skewed curve can be used to describe it. However, with the GO addition, the share of pores with the size of less than 50 µm decreases, favoring the formation of bigger pores to a higher extent, progressing preferentially in the formation of 50–100 µm-sized pores. Thus, the ratio between pores below 50 and above 50 µm decreased from 5.66 (CHT and CHT/ GO 0.5 wt.%) to 3.6 (CHT/ GO 3 wt.%). Above 100 µm, the pore share changes were insignificant and non-linear with respect to the sample’s GO content. The tendency observed with respect to the bigger pores share proneness to increase favors the formation of more cell-friendly architectures in agreement with the in vivo and in vitro reported data.

### 2.4. Cells Distribution and Morphology When Embedded in BC0.5-BC3

**Figure 4:**
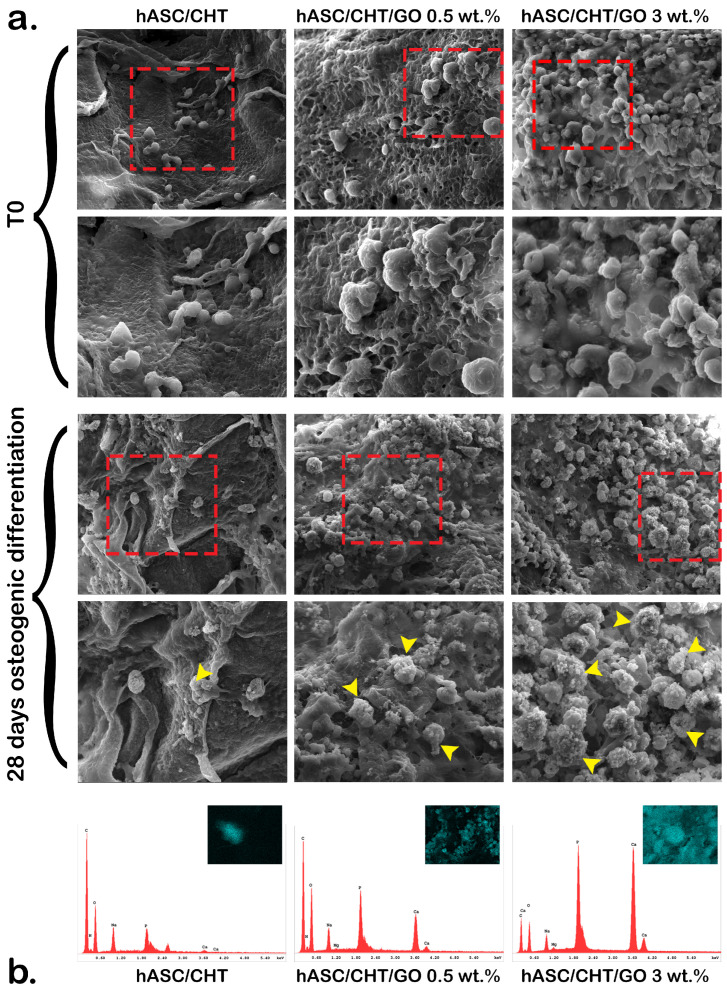
Cell distribution and phenotype in BC0.5–BC3 systems. (**a**) hASC distribution and morphology in the 3D structure of BC0.5–BC3 and the hASC/CHT reference bioconstruct (BC) before and after 28 days of osteogenic differentiation, assessed by SEM; the red box marks the area enlarged below each image and the yellow arrows indicate mineralized deposits in the extracellular matrix (ECM) which was further characterized by EDAX; (**b**) the composition of extracellular matrix secreted by cells after 28 days of osteogenic differentiation, as revealed by energy-dispersive X-ray analysis (EDAX) analysis.

The distribution of hASCs in the porous structure of CHT/GO biomaterials was studied by SEM 48 h after seeding (Figure 4a,T0). Although cell seeding density was the same for all composites and for BC control, there was a lower percentage of cells integrated in the porous CHT network (BC) in comparison to the percentage of cells retained in the GO-containing materials (BC0.5–BC3). A more structured and delineated pore network was found in CHT/GO composites compared to the CHT reference, which has smoother surfaces and a less structured network. The degree of porosity obtained by including different percentages of GO in the structure of the materials probably favored interaction with cells and their retention in the 3D pore network. Additionally, a uniform distribution of hASCs in BC1, BC2, and BC3 versus BC and BC0.5 was observed, which may suggest a GO-dependent cell distribution and adhesion. In our previous studies performed on two-dimensional chitosan/polyvinyl alcohol (CHT/PVA) films enriched with 0.5–6 wt.% GO, we showed a strong tendency of the cells to form oriented groups on the film surface, depending on the GO–distribution material composition [20]. Similarly, the study of Kim et al. [13] highlighted a correlation between the orientation of actin filaments in the cytoskeleton model developed by hASCs and the GO location in the structure of the material.

### 2.5. Evolution of the Osteogenic Differentiation Process in BC0.5–BC3

**Figure 5:**
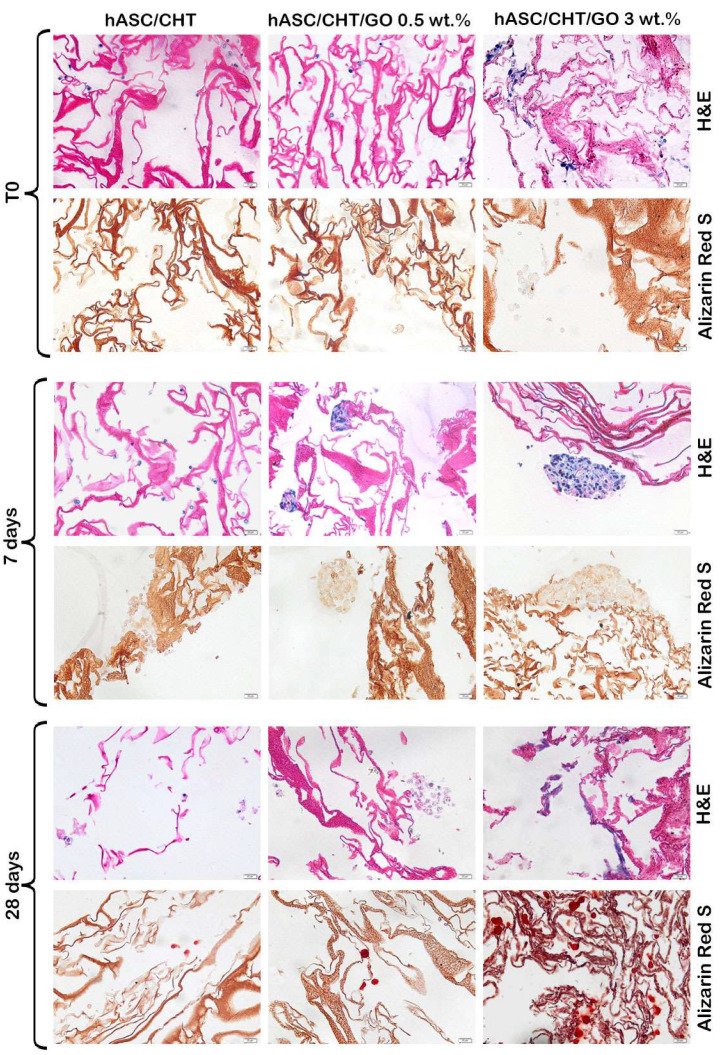
Histological evaluation of osteogenic differentiation. Microtome sections of BC0.5, BC3, and BC stained with hematoxylin-eosin (for morphology) and with Alizarin Red S (to highlight mineral deposits). Scale bar 20 µm.

**Figure 6:**
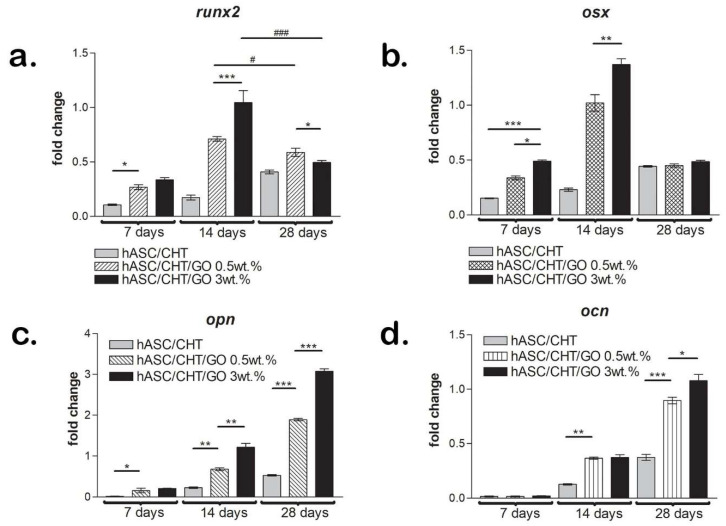
Gene expression of osteogenic specific markers—*runx2*, *osx*, *opn,* and *ocn*. (**a**–**d**) *runx2*, *osx*, *opn*, and *ocn* profiles of gene expression obtained by qPCR in BC, BC0.5, and BC3 after 7, 14, and 28 days of differentiation of hASCs in pro-osteogenic conditions. */# *p* < 0.05; ** *p* < 0.01; ***/### *p* < 0.001. Fold change in qPCR data analysis was determined by 2^−ΔΔCt^ method.

**Figure 7:**
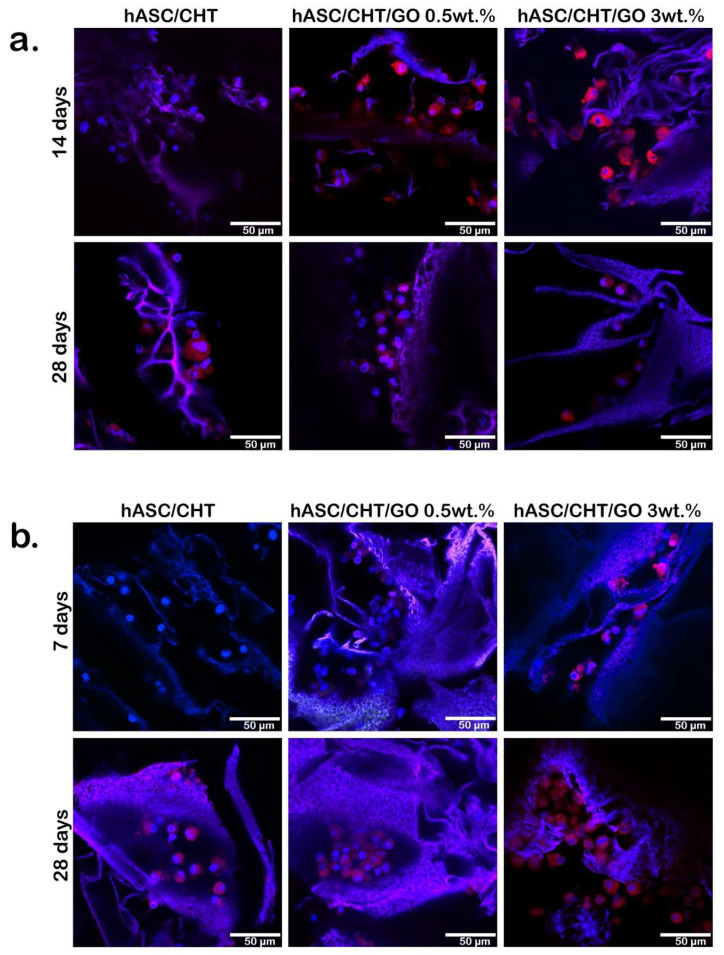
Protein expression of osteogenic specific markers Osx and Opn. (**a**) Osx and (**b**) Opn protein expression as revealed by confocal microscopy in BC, BC0.5, and BC3 up to 28 days of differentiation in pro-osteogenic conditions. The nuclei are labeled in blue with DAPI and Osx/Opn is labeled in red. The scale bar is 50 μm.

CHT/GO and CHT reference materials were exposed to osteogenic inducers over a 28-day period during which the evolution of the hASCs differentiation process in the 3D microenvironment was assessed at multiple levels: (1) morphologically, by SEM (Figure 4a, 28 days osteogenic differentiation); (2) analysis of elements in the extracellular matrix by energy dispersive X-ray analysis (EDAX) (Figure 4b); (3) histologically, to highlight mineral deposits specific for osteogenesis (Figure 5), (4) at the gene level by monitoring the gene expression of early and late osteogenic markers (Figure 6), and (5) at the protein level by highlighting the protein expression of osteogenesis biomarkers (Figure 7).

#### 2.5.1. Bone-like Cell Phenotype and Extracellular Matrix Production in BC0.5–BC3

The morphology of BC0.5–BC3, as well as hASC distribution in the CHT/GO scaffolds after 28 days of osteogenic differentiation, was evidenced by SEM (Figure 4, 28 days osteogenic differentiation), comparatively to the initial culture (T0). The same observations made 48 h after seeding were maintained during the differentiation, namely a smaller percentage of cells identified in BC and BC0.5, and a higher amount of cells retained in BC3. After 28 days of osteogenesis, cells exhibited a round morphology similar to osteoblast-like cells and presented deposits on or around their surface (Figure 4a, marked by arrows). The density of these deposits was found to increase proportionally to the percentage of GO in the material structure.

To verify the chemical nature of the deposits observed through electron microscopy studies, an EDAX was performed for BC0.5, BC3, and BC. The EDAX results showed the presence of calcium and phosphorus in the analyzed samples, thus proving the mineral nature of the deposits present, particularly in BC3 extracellular matrix. These observations led to the hypothesis that hASCs grown in CHT/GO materials developed a similar morphology to osteoblast-like cells and had the ability to secrete bone-specific extracellular matrix after 28 days of osteogenesis.

#### 2.5.2. Evaluation of Osteogenic Differentiation by Histological Staining

H&E histological analysis (Figure 5) demonstrated that the dynamic seeding of the BC0.5–BC3 with cells is time-dependent, with a maximum deposition at 28 days. Addition of GO facilitated and enhanced homogenous colonization of the scaffold pores, followed by abundant cell secreted neo-matrix deposition, while CHT scaffolds hosted cells mainly at the periphery. The greatest quantities of cells and extracellular bone matrix (ECM) were present in BC3.

The hASCs cluster at 7 days after seeding, particularly for 3 wt.% GO. Moreover, at 28 days, the aspect of hASCs was totally changed, from spheroidal to osteoblast-like (spindle-shape) morphology. The Alizarin Red S staining confirmed the osteogenic differentiation, and an abundant calcium-rich osteogenic matrix for BC0.5–BC3 was observed, particularly for BC3.

#### 2.5.3. Evaluation of Osteogenic Markers Gene Expression

The development of the osteogenesis process in BC0.5 and BC3 systems was comparatively evaluated by qPCR versus control, focusing on two early-expressed markers *runx2* and *osx* and two late-activated genes in the osteogenic signaling pathway—*opn* and *ocn*.

The *runx2* transcription factor was detected at the gene level, starting from 7 days post-osteogenic induction in all three systems with statistically higher expression (*p* < 0.05) for biomaterials containing GO than for the control (Figure 6a). After 14 days of hASC differentiation under pro-osteogenic conditions, *runx2* was significantly higher (*p* < 0.001) in hASCs grown in contact with 3 wt.% GO versus 0.5 wt.% GO, which suggests a potential role of GO in activating the differentiation process. In contrast, the *runx2* level was approximately constant between 7 and 14 days in BC, suggesting late osteogenesis activation in the absence of GO. Once most of the cells were engaged in the osteogenic differentiation pathway, *runx2* presence was no longer required and its expression decreased. This shift in the expression profile was better observed in the case of hASCs differentiated in CHT/GO 3 wt.% scaffold (*p* < 0.001), where the level of *runx2* decreased by half between 14 and 28 days of osteogenic differentiation. In contrast, the *runx2* level in BC0.5 recorded a slight decrease (*p* < 0.05) over the same time frame, suggesting a slower differentiation process in this system. In BC, *runx2* expression registered an increasing profile up to 28 days of differentiation, probably due to slow osteogenesis in hASCs cultivated in a scaffold lacking GO.

Analysis of *osx* expression during the 28 days of in vitro induced osteogenesis generated a profile similar to that for *runx2*, with both factors being upstream activators of the osteogenic signaling pathway and therefore considered early markers of the differentiation process (Figure 6b). The gene expression of *osx* was recorded for the first time after 7 days of initiation of differentiation, at levels significantly higher than those measured for *runx2*. Thus, from 7 days of differentiation, there was a statistically significant difference (*p* < 0.001) between the *osx* level quantitated in BC3 and the *osx* level in BC. Also, *osx* expression was significantly higher in the presence of 3 wt.% GO than in the presence of 0.5 wt.% GO at 7 days after induction of differentiation. After 14 days of osteogenesis, the primary *osx* transcript was detected at approximately three-fold higher levels in GO-containing systems, maintaining the statistically significant difference (*p* < 0.01) in favor of BC3, unlike the reference bioconstruct where the *osx* expression was similar to that detected at 7 days post-induction. A significant decrease (*p* < 0.001) of *osx* expression levels between 14 and 28 days of differentiation to levels similar to those recorded at 7 days post-induction was also observed. After 28 days of differentiation under pro-osteogenic conditions, gene expression of *osx* was similar in all three systems studied, with no significant differences.

Late markers of the osteogenesis process—*opn* and *ocn*—were evaluated as indicators of the extracellular matrix secreted by the bone-like differentiated cells. An increasing profile of gene expression for *opn* and *ocn* was detected up to 28 days of differentiation under pro-osteogenic conditions by qPCR. After 7 days from initiation of differentiation, small levels of *opn* were detected in GO-containing systems (*p* < 0.05), while in BC gene expression was positive only after 14 days of osteogenic differentiation (Figure 6c). At 14 days post-induction, the *opn* level was approximately 3-fold higher in the cells grown in contact with BC0.5 (*p* < 0.01) and about 5-fold higher in the BC3 system (*p* < 0.001) than in control. In contrast, the expression levels detected at 14 days post-induction were similar in BC0.5 and BC3 and significantly higher (*p* < 0.01) than the expression in BC (Figure 6d). After 28 days of osteogenic differentiation, *opn* and *ocn* primary transcript levels increased significantly compared to those registered 14 days post-induction, confirming active osteogenesis in cells grown in contact with CHT/GO materials. After 28 days of differentiation under pro-osteogenic conditions, the *opn* and *ocn* primary transcripts were detected at significantly higher levels (*p* < 0.001) in BC0.5 and BC3 than in the control, suggesting the positive effect of GO in bone differentiation.

#### 2.5.4. Protein Expression of Osteogenic Markers in BC0.5-3

In order to confirm the conclusions drawn from the evaluation of the gene expression of osteogenic markers, we analyzed the protein expression of Osx and Opn by confocal microscopy during the 28 days of differentiation, in pro-osteogenic conditions.

At the protein level Osx was expressed earlier, starting 7 days post-osteogenic induction in the BC0.5–BC3 systems, whereas Osx was firstly expressed at 14 days post-osteogenic induction in BC, at very low levels (Figure 7a). After 14 days of differentiation under pro-osteogenic conditions, Osx was highly expressed in BC3. The increased protein expression of Osx in this system correlates with the gene expression levels quantified by qPCR. At the primary transcript level, the highest expression level was observed after 14 days of osteogenic differentiation, similar to the observations made by confocal microscopy. The protein expression profile of Osx recorded a decrease from 14 to 28 days in BC3, similar to the gene expression profile. Same observations were obtained for BC0.5, but to a lower extent. In contrast to the GO-containing systems, Osx expression reached the highest levels only after 28 days of differentiation under pro-osteogenic conditions in the BC reference system, suggesting that osteogenesis progressed in this system with a lower yield or that a smaller proportion of cells from the total seeded amount was successfully induced towards the osteogenic lineage.

In the case of Opn, its expression was identified starting with 7 days of osteogenic differentiation only in BC3 (Figure 7b), and an increased accumulation of the Opn level was observed over the 28 days of differentiation. This demonstrated the efficient osteogenesis development in cells exposed to GO-containing materials and the ability of hASCs to differentiate to mature osteocytes with extracellular matrix secretion capacity. From the point of view of BTE, this aspect is extremely important for the efficient regeneration of bone tissue by producing functional, not inert tissue. In the presence of 0.5 wt.% GO, Opn developed an increasing profile over the 28 days of osteogenic differentiation, but to a significantly lower level than in BC3. This observation suggests the potential role of GO in guiding osteogenic differentiation of hASCs and the potential use of GO in BTE.

The potential of hASCs for differentiation in contact with GO-containing materials was also studied by Kim et al. [13], and osteogenesis evolution on GO films was also confirmed by histological staining with Alizarin Red S. Our results support the same observations, showing both that hASCs were able to differentiate to bone lineage and that GO favored this process. Additionally, supporting evidence [1] showed good biocompatibility of GO materials and acceleration of osteogenic differentiation in human mesenchymal stem cells up to a rate comparable to that of bone morphogenetic protein 2 (BMP-2) induced differentiation.

### 2.6. In Vivo Evaluation of Bone Regeneration Using CHT/GO Materials

Mouse models with a calvaria bone defect received an implant with CHT/GO scaffolds in order to evaluate the efficiency of these materials for bone tissue reconstruction. To assess the progress of bone formation, the Osx osteogenic marker was investigated at both the gene and protein expression level. Considering that Osx is an early osteogenic marker, this evaluation had the purpose of confirming osteogenic process activation in vivo. Additionally, an extensive study has been carried out to investigate the behavior of CHT/GO scaffolds in vivo when implanted in a bone defect [21].

**Figure 8:**
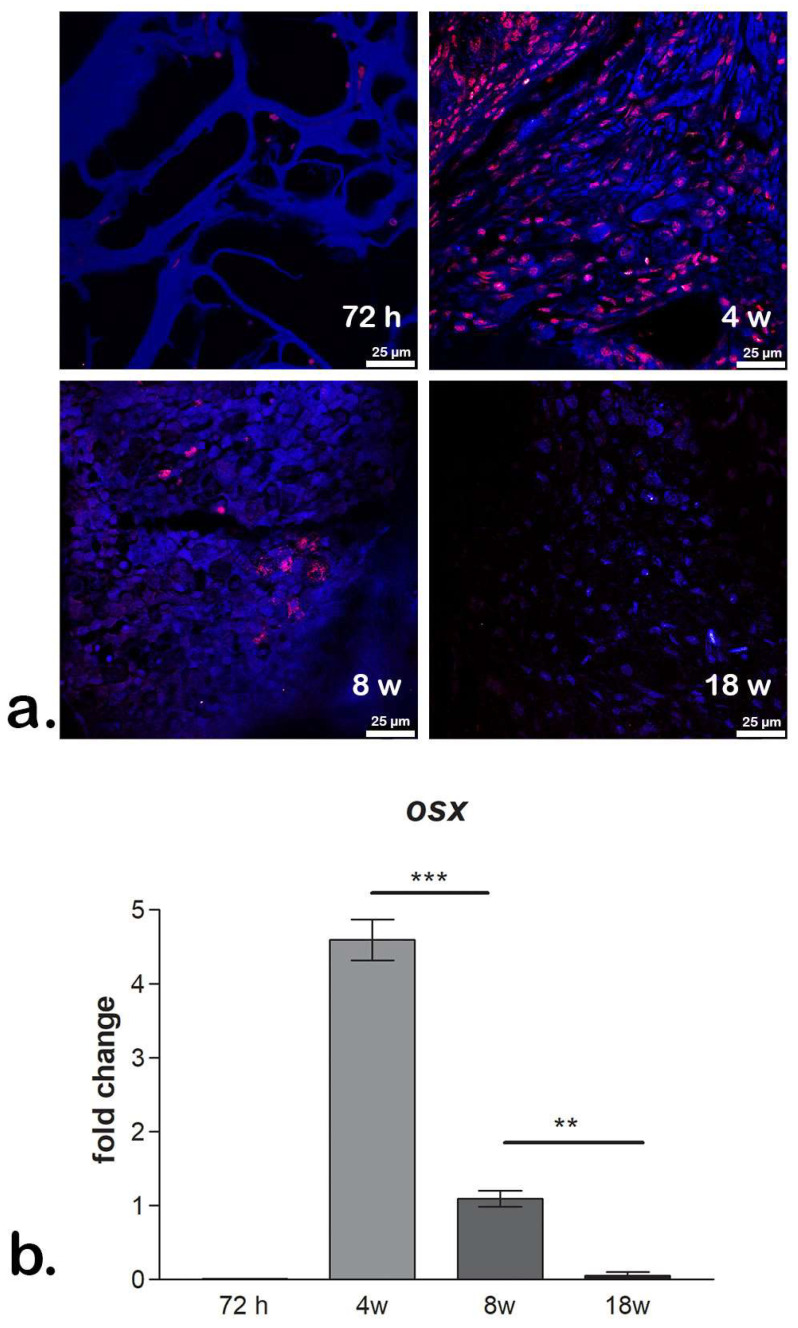
In vivo confirmation of osteogenesis activation. (**a**) Osx levels of protein expression in vivo after CHT/3 wt.% GO material implantation in mouse calvaria bone defect; (**b**) *osx* gene expression profile during 18 weeks post-implantation. ** *p* < 0.01; *** *p* < 0.001. Fold change in qPCR data analysis was determined by 2^−ΔΔCt^ method.

The highest levels of Osx were found 4 weeks after implantation on all composites, with a trend of decreasing in time, up to 18 weeks (Figure 8). Among all compositions, the highest levels of Osx were found in the tissue developed in contact with CHT/GO 3 wt.%, as compared to CHT/GO 0.5 wt.% and to control CHT. Confocal microscopy data (Figure 8a) confirmed bone formation process activation by increased Osx levels 4 weeks after the CHT/3 wt.% GO scaffold was implanted.

Up to date, there is a low number of in vivo studies evaluating the ability of graphene-based nanomaterials to induce and support the production of functional de novo bone tissue [22,23,24,25]. The results of these studies are now also supported by our results.

Based on several studies, GO has the ability to induce by itself the osteogenic differentiation process, but the molecular mechanism underlying this ability has not yet been elucidated. With the purpose of evaluating molecular events taking place during periodontal ligament stem cell differentiation to bone cells in contact with bidimensional and tridimensional graphene-based substrates, Xie et al. [26] showed that a combination of physical and chemical properties of graphene act synergistically to control the osteoinductive effect of graphene.

This section may be divided by subheadings. It should provide a concise and precise description of the experimental results, their interpretation, as well as the experimental conclusions that can be drawn.

## 3. Discussion

Human adipose-derived stem cells have been intensively studies in the last decades for their properties and potential of differentiation and have been established as ideal tools for regenerative medicine and tissue engineering (TE) practices, particularly in the case when the cells are used for the benefit of the same patient that they were isolated from [27]. Bone tissue repair or reconstruction is one of the most widely studied TE applications of hASCs, with positive results both in animal models [28] and in patients [29,30], in an either scaffold-free or scaffold-dependent manner. 

Biomaterials designed for TE have an impact on the cellular component at several levels—the contact modulates cell adhesion, cell proliferation, and even differentiation of stem cells by a chemical composition, physical and mechanical properties, and by a certain microstructural pattern [31,32]. Certain polymers that resemble the natural extracellular matrix (ECM) display greater advantages than others in supporting cell growth and differentiation processes. For instance, collagen, which is naturally found in ECMs, is able to interact with the stem cells via integrin binding [3,33], thus creating the necessary microenvironment for cells to proliferate and differentiate. Similarly, chitosan, a marine polysaccharide, exhibits anti-microbial and non-toxic properties and is known to accelerate the wound healing process, thus contributing to the regeneration of injured tissues [34,35]. Therefore, chitosan-based biomaterials have been widely studied and proved to have beneficial results in TE [36]. However, studies in the last years have shown that composites based on chitosan are actually even better for tissue reconstruction applications. 

Another well-investigated material is graphene and its derivatives, a nanostructured material with great potential in/for tissue engineering applications. Functional groups such as carboxyl, carboxylate, carbonyl, and hydroxyl present on GO’s surface ensure a better interaction with serum proteins and improve cell adhesion, proliferation, and stem cells differentiation towards multiple lineages [37,38,39]. The encapsulation of GO in different materials, such as polyethylene glycol diacrylate (PEGDA) hydrogel, in a previous study by Noh et al. showed promising results in terms of enhanced cell attachment and improved hASC differentiation towards bone lineage, proving GO’s potential for BTE applications.

In a study similar to ours by Kim et al. [13], chitosan materials incorporated with reduced GO were seeded with human mesenchymal stem cells. GO-enriched materials increased cell-cell and cell-substrate interactions compared to chitosan materials and moreover, they promoted osteogenic differentiation even in the absence of osteogenic inducers. Also, a study by Ruan et al. [40] validated in vitro and in vivo another material based on carboxymethyl chitosan and GO for future applications in BTE. A versatile material for tissue reconstruction proved to be a composite based on cellulose acetate, carbon nanotubes and GO, which generated low cytotoxicity and increased profile of bone differentiation markers when used in combination with hASCs [41]. 

The potential of hASCs for differentiation in contact with GO-containing materials was also studied by Kim et al. [13], and osteogenesis evolution on GO films was also confirmed by histological staining with Alizarin Red S. Our results support the same observations, showing both that hASCs were able to differentiate to bone lineage and that GO favored this process. Additionally, supporting evidence [1] showed good biocompatibility of GO materials and acceleration of osteogenic differentiation in human mesenchymal stem cells up to a rate comparable to that of bone morphogenetic protein 2 (BMP-2)-induced differentiation. 

In this context, we previously carried out an extensive study to investigate the behavior of our CHT/GO scaffolds in vivo when implanted in a bone defect [21]. The materials were implanted in mouse models with calvaria defects and bone regeneration was monitored for up to 18 weeks. Histopathological and scanning electron microscopy (SEM) analysis of the implants revealed larger amounts of new bone in the CHT/GO-filled defects. In addition, the level of bone markers bone morphogenetic protein (BMP) and Runx-2, as well as the alkaline phosphatase activity, showed that GO promoted bone differentiation efficiently and proportional to its concentration in the composite materials. Furthermore, CHT/3 wt.% GO presented a significant increase in the number of Opn and Ocnpositive cells when compared to CHT/0.5 wt.% GO or CHT alone, confirming that that GO facilitates cell infiltration and differentiation of osteoprogenitor cells to the osteogenic lineage dependent on time frame and GO concentration.

## 4. Materials and Methods 

All methods described below were performed in accordance to current research guidelines and regulations.

### 4.1. Material Preparation

Scaffolds based on CHT and GO were prepared as previously described [21,42]. Briefly, 0.5–3 wt.% GO was incorporated within CHT biopolymer matrix. Pure chitosan scaffolds were used as controls for the experiments.

### 4.2. Material Characterization by MicroCT Analysis

From each sample, two specimens (approximately 6 × 6 × 6 mm) were acquired and used without further treatment. For the micro-computer tomography analysis, Bruker microCT 1172 high-resolution equipment was used. The specimens were scanned without filter, at a source voltage and current intensity set for 45 kV and 200 µA, respectively, and an exposure per frame of 300 ms. The scans were performed applying 180° rotations of the sample, with a rotation step of 0.15°. Each slice capture resulted from the mean of five average frames. Throughout the sample set, the image pixel size corresponded to 5.13 µm. Bruker NRecon software was employed to reconstruct the tomograms from the raw data. In terms of parameters, 25-beam hardening correction set to 25, 17-ring artefact reduction, and smoothing to 1 were used. Reconstructed tomograms were rendered in CTVox (Bruker), while sample analysis was performed in CTAn software (Bruker). For each composite, six cylindrical volume of interest (VOI) datasets were extracted, three from each specimen. The VOIs were constrained in terms of diameter (4 mm) and height (300 slices). For the 3D analysis in CTAn, the VOIs were subjected to an image processing task, thresholding to singularly separate the specimen walls from its pores, despeckling for the removal of remnant scanning artefacts, and 3D analysis for the quantification of specific surface, total porosity, structure separation, and structure thickness. Numerical results are reported as mean values recorded for the six VOIs of each composite formulation with standard deviation (± SD).

### 4.3. Cell Culture

Human adipose-derived stem cells (hASCs) used for this study were purchased (StemPro, ThermoScientific, Waltham, MA, USA) and cultured up to passage 4 in the recommended cell culture media supplemented with 10% fetal bovine serum (FBS, Life Tehnologies, 

Bleiswijk, Netherlands) and 1% antibiotics (Sigma-Aldrich, Darmstadt, Germany), in standard culture conditions (37 °C, humidity and 5% CO2). All in vitro experiments including hASCs were approved by the Ethics Committee of the University of Bucharest (approval number 153 on 24 August 2017) and are in accordance to the current regulations and guidelines for adult stem cells handling.

hASCs in the fourth passage were seeded on the surface of all scaffolds and were allowed for 24h to diffuse through the network of pores in the scaffolds to populate the entire volumes. The resulting cell-3D scaffold systems were further called “bioconstructs” (BCs) and referred as follows: the hASC/CHT control system (BC), the hASC/CHT/0.5 wt.% GO system (BC0.5), the hASC/CHT/1 wt.% GO system (BC1), the hASCs/CHT/2 wt.% GO system (BC2), and the hASCs/CHT/3 wt.% GO system (BC3).

### 4.4. In Vitro Experiments

#### 4.4.1. Biocompatibility Assays

All materials were tested for biocompatibility in contact with hASCs during one week of standard culture conditions, namely after 2, 4, and 7 days of culture. Cell viability and proliferation were tested both quantitatively by MTT test (3-(4,5-dimethylthiazol-2-yl)-2,5-diphenyltetrazolium bromide, Sigma Aldrich, Darmstadt, Germany) and qualitatively by fluorescence microscopy using Live Dead assay (ThermoScientific, Waltham, MA, USA). LDH assay (Tox7, Sigma-Aldrich, Darmstadt, Germany) was employed to quantify the percentage of dead cells in the 3D cultures. MTT and LDH assay results were read by spectrophotometry at 550 nm and 490 nm, respectively.

#### 4.4.2. Cytoskeleton Assessment

F-actin filaments developed by hASCs in contact with each material were studied by confocal microscopy using a Carl Zeiss LSM710 laser-scanning microscope. hASC morphology and cytoskeleton fibers distribution in contact with CHT/GO scaffolds were studied at 48 h post seeding. In order to fluorescently label F-actin, constructs were fixed with 4% paraformaldehyde (PFA) for 8 h and permeabilized with 2% BSA/0.1% Triton X-100 solution at 4 °C. Next, the constructs were incubated 4 h at 37 °C with Phalloidin-FITC (Sigma-Aldrich, Darmstadt, Germany). Cell nuclei were stained with DAPI for 30 min. Carl Zeiss Zen 2010 software version 6.0 was used for image acquisition and analysis.

#### 4.4.3. Cell Morphology Before and During Differentiation by Scanning Electron Microscopy (SEM)

The samples (implanted calvaria and surrounding tissue) were mounted on conductive pin stub using on both sides adhesive carbon discs. The ex vivo samples were gold metallized using an Agar sputter coater with a deposition of 3 nm thickness three times [43]. The analyzed parameters were HV mode, ETD, 5–20 kV, and 100–300× magnification for a general overview image and higher for surface and morphology evaluation. Examination, image analysis, and EDAX were conducted on a FEI Quanta 250 microscope. 

#### 4.4.4. Histological Evaluation of Bone Differentiation

Here, 4% formaldehyde in phosphate buffer solution (PBS) was used to fix the samples, which were dehydrated in a graded series of ethanol and embedded in paraffin blocks. Samples sections (5 μm) were deparaffinized, hydrated in a graded series of alcohol solutions, and stained with H&E for cell colonization analysis throughout the scaffolds and Alizarin Red S to visualize the calcium-rich cell matrix at T0, 7 days, and 28 days of osteogenic differentiation. Light microscopic images were taken with Olympus Bx 43 microscope (Olympus, Tokyo, Japan) equipped with Olympus XC30 camera.

#### 4.4.5. Bone Markers Gene Expression Evaluation by Quantitative PCR (qPCR)

hASC/CHT/GO bioconstructs were cut into fragments and the total RNA was isolated using TRIzol Reagent (Invitrogen, Foster City, CA, USA) in accordance with the manufacturer’s instructions. After the isolated total RNA was tested for purity and concentration on the NanoDrop spectrophotometer (ThermoScientific, Waltham, MA, USA), and for integrity on the BioAnalyzer 2100 (Agilent Technologies, Waldbronn, Germany), total cellular RNA was reverse-transcribed to cDNA using iScript cDNA Synthesis kit (BioRad, Hercules, CA, USA). Quantitative PCR was performed to assess gene expression levels for *runt-related transcription factor 2* (*runx2*), *osterix (osx), osteopontin (opn*), and *osteocalcin (ocn*)-specific osteogenic markers using Viia 7 equipment (ThermoScientific, Waltham, MA, USA) and SYBR Green method of detection. TATA Binding Protein (TBP) was used as the reference gene and was assessed in the same experimental conditions. Fold change in qPCR data analysis was determined by the 2^−ΔΔCt^ method.

#### 4.4.6. Bone Marker Protein Expression Evaluation by Confocal Microscopy

Osterix and osteopontin bone-specific markers were assessed by confocal microscopy after 7 and 28 days of osteogenic differentiation. Constructs were fixed with 4% PFA for 8 h and permeabilized with 2% BSA/0.1% Triton X-100 solution at 4 °C. Next, samples were incubated 4 h at 37 °C with mouse polyclonal anti-Osx and goat polyclonal anti-Opn (Santa-Cruz Biotechnology, Heidelberg, Germany) antibodies. The bioconstructs were further incubated in tetramethylrodamine-5,6-isothiocyanate (TRITC) conjugated goat anti mouse and fluorescein isothiocyanate (FITC) conjugated rabbit anti goat secondary antibodies solutions for 1 h (Santa-Cruz Biotechnology, Heidelberg, Germany). Nuclei were stained with DAPI for 30 min. Finally, samples were visualized in confocal microscopy.

### 4.5. In Vivo Experiments

#### 4.5.1. Animals and Experimental Design

For the in vivo experiment CD1 mice were used. Experimental procedures were approved by the Ethics Committee for Research of the Vasile Goldis Western University of Arad (approval no.131, approved on 13 December 2018 for research activities involving animal models in project GRABTOP, P_37_221/2015, SMIS code 108117and are in accordance with current guidelines and procedures established for animal studies. The mice were randomly divided as follows: control group*,* empty defect; CHT group, chitosan scaffolds implanted; CHT/0.5 wt.% GO group, chitosan, 0.5 wt.% graphene oxide implanted; CHT/3.0 wt.% GO group*,* chitosan, 3.0 wt.% graphene oxide implant. Before surgical intervention, all the scaffolds were sterilized for 20 min by UV.

All mice were anesthetized with 100 mg/kg b.w. ketamine hydrochloride/10 mg/kg b.w. xylazine hydrochloride, and 5-mm full-thickness craniotomy defects were achieved using a 3.5-mm drill (Super NP5, Korea) under constant saline irrigation [21]. All mice survived after the surgery and were housed in individual cages under constant conditions.

The mice were euthanatized by an overdose of anesthetic after 72 h or 4, 8, or 18 weeks (*n* = 10/each group and per time point). All the implants were harvested for immunofluorescence and qPCR studies on bone marker expression, using the same protocols as described above.

#### 4.5.2. Statistical Data Analysis

All data were statistically analyzed using GraphPad Prism 3.03 Software (GraphPad Software Ink., San Diego, CA, USA), one-way ANOVA, Bonferroni test, considering *p* < 0.05 as significant. The experiments were performed with *n* = 3 biological replicates and each data set is presented as the average of three replicates (mean ± standard deviation). 

## 5. Conclusions 

Chitosan-based scaffolds, improved with 0.5–3 wt.% GO, proved to be biocompatible in terms of low cytotoxicity, good cell viability, and increased cell proliferation during one week of culture. Once synthesized, microCT results confirmed the development of highly porous scaffolds, with open interconnected porosity consisting of both macro- and micro-pores. The GO addition determined the formation of more ordered morphologies with higher total porosity and greater surface available for cell attachment. These features reflected very efficient cell adhesion to the materials, dependent on the GO concentration in the scaffold. CHT/GO scaffolds proved to support in vitro hASC osteogenic differentiation for 28 days, as well as in vivo bone repair in mouse models for 18 weeks. The chitosan scaffold improved with 3 wt.% GO (CHT/GO 3 wt.%) revealed the highest levels of osteogenic markers both in vitro and in vivo and thus can be considered as a promising solution for further bone tissue engineering approaches.

## Figures and Tables

**Table 1:** Spreadsheet of the readings of total porosity (T.Po), structure thickness (St.Th), and specific surface (Sp.S), calculated as the ratio of object surface and object volume. The numbers are the average values of the 3D analysis carried out in CTAn for six distinct proportionate volumes of interest (VOIs) per composite sample.

Sample	T.Po (%)	St.Th. (µ)	Sp.S (µ^−1^)
CH	74 ± 1.49	16.8 ± 2.64	0.195 ± 0.03
CH/0.5 wt.% GO	71.7 ± 1.89	20.1 ± 5.54	0.169 ± 0.05
CH/3 wt.% GO	76.4 ± 4.82	20.3 ± 4.32	0.181 ± 0.08
