# Correction: Dinescu et al. Graphene Oxide Enhances Chitosan-Based 3D Scaffold Properties for Bone Tissue Engineering. *Int. J. Mol. Sci.* 2019, *20*, 5077

**DOI:** 10.3390/ijms27052424

**Published:** 2026-03-06

**Authors:** Sorina Dinescu, Mariana Ionita, Simona-Rebeca Ignat, Marieta Costache, Anca Hermenean

**Affiliations:** 1 Department of Biochemistry and Molecular Biology, University of Bucharest, Splaiul Independentei 91-95, 050095 Bucharest, Romania; simona.ignat@unibuc.ro (S.-R.I.);; 2 Advanced Polymer Materials Group, University Politehnica of Bucharest, Gh. Polizu 1-7, 011061 Bucharest, Romania; mariana.ionita@polimi.it; 3 Institute of Life Sciences, Vasile Goldis Western University of Arad, 86 Rebreanu, 310414 Arad, Romania; anca.hermenean@gmail.com

## Correspondence

In the original publication [1], Prof. Marieta Costache was listed as the corresponding author. Following her passing in February 2024, an update to the correspondence information is required. Dr. Sorina Dinescu is now designated as the corresponding author. The scientific conclusions are unaffected. The original publication has been updated.

## Error in Figure

In the original publication [1], there was an error in Figure 4. The image corresponding to the hASC/CHT composite at 28 days of differentiation was incorrectly selected and corresponded to the hASC/CHT/GO 0.5 wt.% composite. This error occurred due to inadvertent selection of an image from the incorrect image folder during figure preparation. The corrected Figure 4 appears below; the figure caption remains unchanged. The authors state that the scientific conclusions are unaffected. This correction was approved by the Academic Editor. The original publication has also been updated.

## Figures and Tables

**Figure 4:**
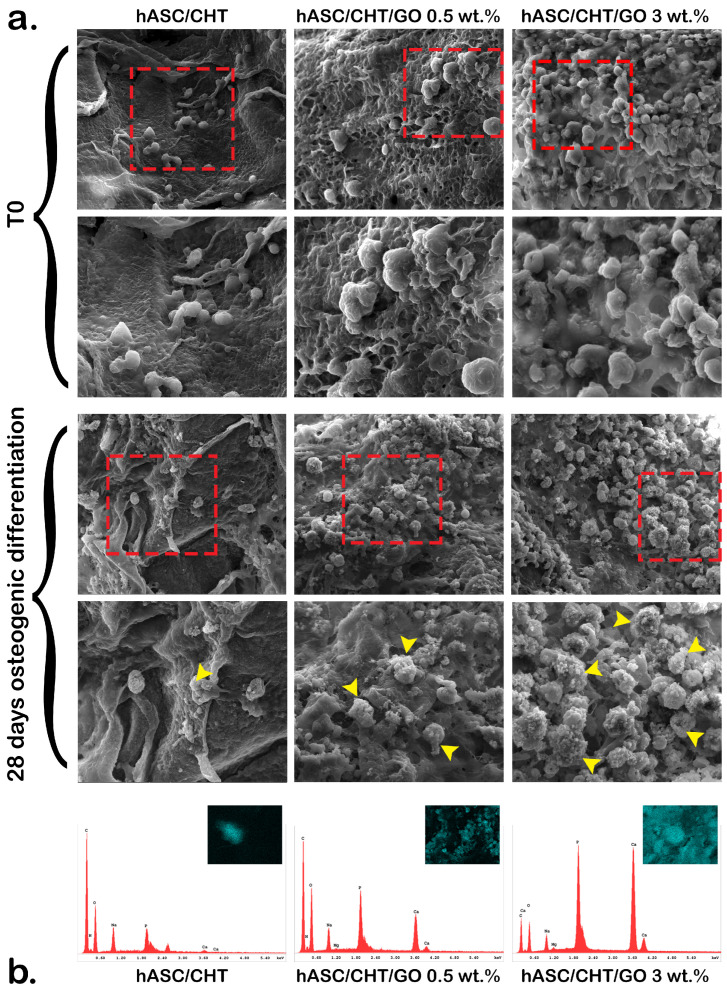
Cell distribution and phenotype in BC0.5–BC3 systems. (**a**) hASC distribution and morphology in the 3D structure of BC0.5–BC3 and the hASC/CHT reference bioconstruct (BC) before and after 28 days of osteogenic differentiation, assessed by SEM; the red box marks the area enlarged below each image and the yellow arrows indicate mineralized deposits in the extracellular matrix (ECM) which was further characterized by EDAX; (**b**) the composition of extracellular matrix secreted by cells after 28 days of osteogenic differentiation, as revealed by energy-dispersive X-ray analysis (EDAX) analysis.
